# Supplementary material for: Adherence to an overweight and obesity treatment: how to motivate a patient?
Source: PeerJ. 2014 Jul 29;2:e495. doi: 10.7717/peerj.495 (PMC4121588; doi:10.7717/peerj.495)
Supplement: Supplemental Information — http://dx.doi.org/10.6084/m9.figshare.1032566 [file peerj-02-495-s001.docx]

. Isaac Kuzmar. [fig**share**](http://figshare.com/).

Retrieved 18:42, Jun 27, 2014 (GMT)

<http://dx.doi.org/10.6084/m9.figshare.1032566>- See more at: http://figshare.com/preview/_preview/1032566#sthash.YYSJTczk.dpuf
